# The spatialization of decent work and the role of employability empowerment for minority ethnic young people in emerging economies

**DOI:** 10.1371/journal.pone.0297487

**Published:** 2024-02-27

**Authors:** Tony Wall, Nga Thi Hang Ngo, Scott Foster, Phuong Minh Luong, Tien Thi Hanh Ho, Ann Hindley, Peter Stokes

**Affiliations:** 1 Liverpool Business School, Liverpool John Moores University, Liverpool, United Kingdom; 2 Tay Bac University, Son La, Vietnam; 3 Vietnam Japan University (Vietnam National University), Hanoi, Vietnam; 4 Phu Xuan University, Hue, Vietnam; 5 Leicester Castle Business School, De Montfort University, Leicester, United Kingdom; National Cancer Center Institution for Cancer Control, JAPAN

## Abstract

Global rises in precarious labour conditions have prompted further empirical work in Decent Work, a special category of employment characterised by equitable pay, treatment, and healthy working conditions. Despite this, research has tended to be conducted in developed countries with privileged groups such as those with typical working arrangements and rely on psychologically framed individual characteristics to explain marginalising factors. We propose a more sociologically framed, spatialised perspective on Decent Work which posits that marginalising factors are spatially variable and determined but moderated by employability empowerment. We measure our propositions across three spatially different sites of Vietnam through (1) a survey of minority ethnic students and graduates (N = 1071) and (2) a survey of stakeholders involved in the recruitment and employment of this group (N = 204). We find support for most of our propositions and call for more spatialised empirical work in the field of Decent Work.

## Introduction

While the empirical investigation of Decent Work has risen sharply in recent years its theorisation has not progressed beyond a psychological perspective [1]. This is despite the utility of sociological theory in understanding the dynamics of labour markets and (mis)match of the supply and demand of labour, and when in the context of higher education, the sociocultural aspects of employability. To date, empirical work into employability and mobility has highlighted the interactional dimensions of macro factors (such as split labour markets and differential labour market outcomes), meso factors (such as the sociocultural structuration of occupations and the gendered and racialised practices of organisations), and micro factors (such as human capital development). Within the context of Decent Work, however, empirical work fails to embrace spatialised perspectives and so does not account for how mobility dynamics might operate differently across different locational and associated sociocultural places. This focus is arguably obfuscated by a narrow focus in empirical work on developed countries with privileged groups such as those with typical working arrangements and relies on psychologically framed individual characteristics to moderate marginalising factors [2].

In contrast to the extant literature, we examine Decent Work from a spatialised perspective, in the context of minority ethnic young people in an emerging economy. This is a group that, due to the massification of education and wider economic conditions, that is often under- or un- employed [3], but particularly so in the emerging economy of Vietnam [4]. This is a group who are evidenced to face some of the worst employment prospects in the world and are exposed to some of the most precarious work environments (such as the lowest paid work) partly due to racial discrimination [4]. Globally, 21.2% of young people are unemployed or not in education or training, but this figure reaches as high as 40% in some countries [5].

We propose a broader conceptualisation of the psychologically framed individual characteristics which moderate marginalising factors, informed by different expressions of power [6, 7] across different levels of a sociocultural system [8]. As such, we introduce a more sociologically framed, spatialised perspective on Decent Work which posits that employability empowerment (an individual expressing different forms of power to change their employment circumstances) has a role in moderating these marginalising factors, and that these are spatially variable and determined.

This article is structured as follows. The first section highlights some of the ongoing issues and tensions in researching Decent Work. Following this, we propose an alternative perspective to Decent Work which foregrounds contextualisation, driven by an emerging spatialisation perspective, where locational factors and interpretations play a significant part in the mobility dynamics associated with Decent Work. Next, the methods of the study are discussed, outlining the two waves of the study. The results of the study are then discussed in relation to the extant literature to outline new contributions. After outlining the limitations, boundaries, and proposal for future research, the implications of a spatialised approach to Decent Work are outlined in relation to policy and educational institutions.

## Beyond Decent Work as a normative standard

As an international labour standard, Decent Work attempts to provide normative criteria through which to raise global standards of work and employment protection. Empirical work, however, argues that the effectiveness of Decent Work as a normalising instrument continues to be problematic because of governance gaps including an overreliance on compliance in a market [9]. As such, evidence highlights the complex sociocultural challenge of attempting to change and sustain employment opportunities and labour standards across diverse cultural milieu including some of the most disadvantaged communities in the world.

In contrast to Decent Work’s heritage in advocacy, recent empirical work into Decent Work has emerged from a psychological tradition, where a series of predictors have been examined and a scale has been validated across multiple countries [1]. Here, Duffy and colleagues define Decent Work as possessing

1. physical and interpersonally safe working conditions (e.g., absent of physical, mental, or emotional abuse), (b) hours that allow for free time and adequate rest, (c) organizational values that complement family and social values, (d) adequate compensation, and I access to adequate health care [10, p130].

Duffy and colleagues identify four direct predictors of Decent Work, with variable support across studies: economic constraints, marginalisation, work volition, and career adaptability [11]. These macro conditions include the economic status and constraints of individuals and their families, their social capital, as well as wider sociocultural marginalisation [12]. Marginalising conditions include ethnicity, gender, immigrant status, as well as other minority statuses such as age and disability [13, 14].

This psychological work, however, has tended to focus on the typical employment scenarios (e.g. paid work with a single employer) related to standard employment arrangements (e.g. full time) of adults in developed countries such as the United States [2]. Despite the original conception of Decent Work being rooted in raising international labour standards, there remains a “historical focus on higher-income settings and workers with relatively privileged status” [2, p1]. The ambitions for political normalisation and the emergence of a unifying normative, de-contextualised theory about Decent Work remain controversial [15], because they both embrace the homogenous notion of decency across sub-localities, sectors, types of work, or tasks within that work [16] as well as situational or transitional circumstances such as displacement or refugee resettlement [17]. At the same time, evidence suggests that the dynamics associated with economic conditions and marginalisation in emerging economies are different to developed economies; developing economies demonstrate economic and educational exclusion of ethnic minorities and women, and income inequality above average of all OECD countries [18]. Moreover, the dynamics of emerging economies are disproportionately affected climate hazards and climate change, which cause increasingly destructive impacts on economic and health deprivation and inequalities [18].

Emerging economic contexts provide useful empirical sites because the economic growth of industrial sectors is particularly significant to mobility, especially due to the prospects of relative wage or career progression or to escape a precarious work condition [19]. In such contexts, individual behaviour changes: when an economy is perceived to be in a state of growth there is generally a greater sense of predictability and optimism, and so people tend to seek new or better employment opportunities with a view to invest in the future [20]. Conversely, when an economy is seen to be in retraction or harsher more generally, there is more unpredictability, so people adopt shorter time horizons [20].

## Spatialised (exclusionary) dynamics of Decent Work

The theoretical and empirical predictors of Decent Work implicitly reflect established literature that mobility, or the access and participation to jobs or occupations, is shaped by the interactions between macro features (such as the structure of job markets and macroeconomic expansion or turbulence) and micro features (such as individual preferences and resources), and their mediation through race and gender [21]. This more established literature on mobility demonstrates a complex multitude of influences spanning structural (e.g. economic conditions), occupational (e.g. role expectations), organisational (e.g. cultural preferences), work group, personal life, and personality and personal style perspectives [19]. Together, such features can be seen to interact to shape the opportunity structures of jobs and individual preferences and decision making in line with social norms and the desirability of mobility [19].

This proposition is also reflected the extant literature of mobility in the context of higher education, moving beyond the limitations of naïve matching of skills demand-supply, towards the spatially bounded institutional perspective. An institutionalist perspective positions employers as deeply contextualised within wider social fields and structures, influenced by exogenous factors in the wider labour market, comprising the social hierarchies, values, practices, and expectations, that are reflective of particular locations, occupations, sectors, and roles [3]. This perspective asserts the highly socio-culturally specific nature of signals and their interpretation, which can represent the possibility of highly variable employer assessments of value, and employment outcomes, across locations, organisations, and sectors [22]. As such, this socio-culturally complex isomorphic process involves employers interpreting partially symbolic signals for dispositions and competencies [23]. Additional factors which highlight the peculiarities of context include:

*Location*: Employment opportunities are spatially distributed, that is, unevenly distributed across communities [24, 25]. Place-sensitive models now provide more predictive strength to modelling youth labour market integration, because they take into account locational disparities of youth labour markets and complex interactions at the local level [26]. Empirical work now positions group biases and discrimination, developed through participation in education, community, and work, as a function of place [27], and can be reflected in location and *re*location to access work [28].

*Demographics (Ethnicity*, *Gender*, *Age*, *intersectionality)*: Labour market assimilation and split labour market theory explain how race/ethnicity introduces group-level hierarchies which amplify exclusionary processes of social closure and discrimination, negatively affecting the opportunities available for minority ethnic people [13, 29, 30] and penalties such as in lower wages [31]. In terms of gender, there is long-standing evidence base in relation to the way women are disproportionately represented in precarious employment, oppressive working conditions, and unequal terms of payment [32]. Age has also been found to moderate access to Decent Work, partly due to discrimination but also due to younger people being more susceptible to engage in more precarious work [33]. When gender intersects with other characteristics such as age, race or disability, these disparities and inequalities become even more pronounced [32].

*Subject majors*: The subject areas of education have signalling properties [22, 34], but that this signalling variably connects with the occupational and organisational field in which it is expected to be applied, mediating the employment outcome differently across fields [35].

*Type of employment / sources of income*: There are scarring and penalty effects from part-time work as well as unemployment and that this is mediated by localised perceptions of competence and commitment [36]. Similarly, evidence argues that the effects of unemployment extend over a lifetime but are spatially variable [37], and these are pronounced for minority ethnic workers [38].

*Institutional framework initiatives*: The above factors may also be influenced at national and provincial levels through different skills frameworks, empowerment initiatives, growth and productivity plans, support for entrepreneurship, and education policy [39]. However, evidence suggests that initiatives might accelerate employment integration (e.g. the achievement of a job) but can also promote the acceptance of job terms and conditions (such as wage) lesser than through other means [37].

## Employability empowerment across cultural milieu

Whilst spatialised exclusionary factors seemingly shape employability outcomes and aspects of Decent Work, there is evidence which suggests that psychosocial resources can *navigate* the wider contextual parameters or material barriers to change one’s own employment and life decisions [11]. These have been expressed variably as work volition and career adaptability [12], and in broader literature as a synonym for the behavioural tendencies for responding to changing labour market conditions across career stages [40].

However, the literature also highlights young peoples’ critical consciousness [41, 42] as a critical behaviour to “perceive social, political, and economic contradictions, and to take action against the oppressive elements of reality” [43, p36]. As such, it can be understood as a socio-political capacity to liberate oneself from the oppressive conditions of structural group bias and has been demonstrated to shape younger peoples’ orientations towards work as well as their expectations of the sorts of occupations they expect to occupy later in life, thereby acting as an agentic *negotiation* of constraints [44]. Critical consciousness therefore moves from *navigating* to *negotiating* these sociocultural constraints, akin to notions of empowerment [45]. This suggests:

*Hypothesised relationship 1: Spatialised exclusionary factors mediate the relationship between perceived employability empowerment and experience of Decent Work.*

Negotiating socio-cultural parameters moves from psychologically oriented, individual characteristics perspective, towards a sociocultural, ecosystemic perspective, which develops a more complex understanding of wider structural conditions in relation to employability and career development. This broader perspective considers the development and expression of power, in that it is contextualised in social settings, shapes mobility, and acts as a response to discrimination [46]. Here, the expression of empowerment moves beyond, but includes the narrow view of ‘*power over*’, where institutions and their instruments dominate or coerce its citizens to form new ways of thinking or acting, for example, to change their work, employment, or livelihood situation [7]. This is a typical view of power used to express how hegemonic institutions or elites mobilise their power, but there are other expressions often adopted in participatory policy development [6]. Alternative expressions of power have been described as: *power-within* (a person having the self-awareness, self-esteem, and confidence to believe they can change a situation), *power-with* (a person being able to contribute to collective action to change a situation), and *power-to* (a person being able to deliver effective action to learn how to change a situation) [47]. As such, the four expressions of empowerment provide a more holistic typology to describe how power is enacted.

In this broader conceptualisation, an ecosystemic perspective also delineates micro, meso, exo, and macro systems which influence access to, and participation in, circumstances which can affect human development [48]: micro-systems refer to the activities, roles, and interpersonal relationships; the meso-system involves the linkages between two or more of these settings (e.g. family, education, and livelihood contexts); exo-systems refer to the wider events and policies which impact the conditions and resources within those family and education settings; and the macro-system refers to the wider cultural values, beliefs, and customs, and the broad cultural, political, social, and economic climate. When empowerment is conceptualised in these ways in relation to employability, ‘employability empowerment’ is a socio-culturally embedded practice expressed across social settings which variously influence access and participation in Decent Work. From the extant literature discussed above, this includes the various capitals expressed through educational, family and community, and employment contexts.

*Hypothesised relationship 2: Spatialised exclusionary factors mediate the relationship between perceived employability empowerment and the expression of practices in different settings.*

In terms of educational settings, students and graduates are not just learning skills and knowledge but developing social capital to access and engage in opportunities relevant to local labour markets [49]. Educational institutions are also building and framing navigational and negotiational resources in relation to labour markets and lives. For example, research has found that the employment counselling and advice services provided only limited options to young people in low skilled opportunities and in line with gendered role expectations [50]. Students and graduates are also developing these resources in terms of family and community settings, framed by familial roles and expectations as well as highly localised community roles and expectations [51]. Similarly, in terms of employment, the ability to express empowerment practices in workplaces will reflect the possibilities afforded by localised cultural contexts and environments [52]. This includes, for example, hiring, role allocation, pay, reward and compensation, and structuring [53].

*Hypothesised relationship 3: Spatialised exclusionary factors mediate the relationship between contextualised employability practices and experience of Decent Work.*

We conceptualise this broader concept as ‘employability empowerment’. There are two implications of this. First, it expands a view of navigational resources to include negotiational resources to broaden the person-driven focus to include different levels of the sociocultural system. It highlights the possibility that each of these levels have a role in shaping Decent Work outcomes. Second, it recognises that employability develops across contexts, where structural disadvantage shapes the development of resources such as social capital. Educational, community, and employment contexts are noteworthy as they frame the “structure of opportunity” which shapes “learning experiences and affects the translation of interests into career goals and outcomes” [2, p7]. Such a perspective therefore extends the current myopia in the current psychological literature to the “the structure of opportunity, sociopolitical context, and social class” [2, p7].

**Fig 1:**
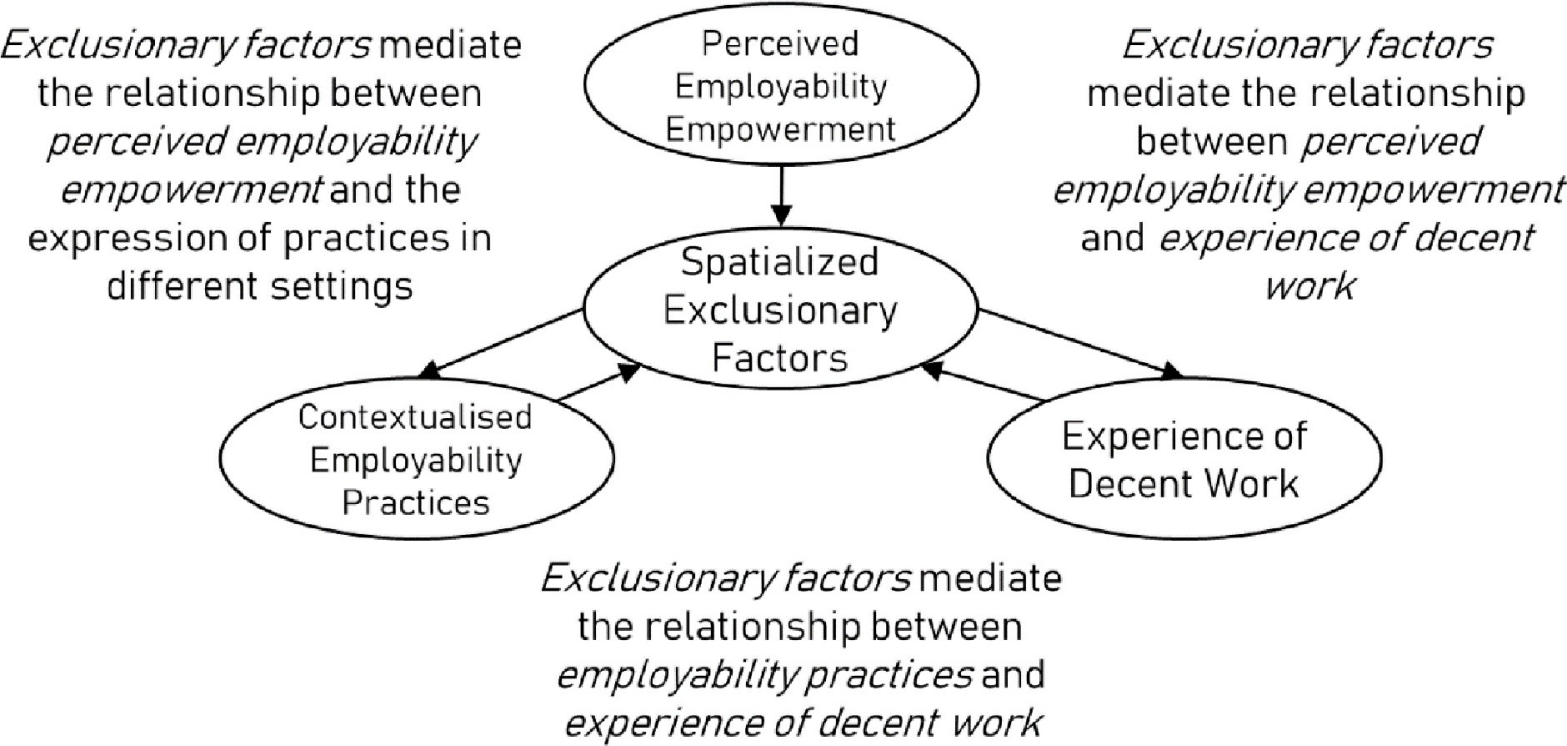
Primary relationships conceptualized in this study.

In sum, the extant literature proposes hypothesised relationships between employability empowerment and Decent Work, expressed through sociocultural contexts and mediated through spatialised exclusionary factors. Specifically, we can hypothesise that spatialised exclusionary factors mediate the expression of employability empowerment across different settings, and that this expression is framed by and reflects local social structures. Given the expression of this empowerment will be cast within local sociocultural schemas, it is reasonable to assume such expressions will be shaped by the same exclusionary factors outlined above. This proposal is summarised by the following broad hypotheses, and in the figure below (Fig 1):

## Methods

### Context of study: Vietnam as an emerging economy

Vietnam has sustained its position as one of the fastest growing economies [4], catalysed by the “Doi Moi” economic reform policy of 1986. Vietnam has dropped down the Global Talent Competitiveness Index, where it is now ranked as 96^th^ out of 132 countries partly due to the relocation of relatively low/unskilled, low-wage manufacturing work from China [54]. The agricultural sector in Vietnam constitutes up to 25% of GDP, over 70% of employment [55], and 86% of Vietnam’s poorest people identify as one of Vietnam’s 54 ethnic minority groups [56]. Ethnic minority households in Vietnam demonstrate “significantly higher rates of deprivation in all dimensions compared to their non-minority counterparts” [57, p161]. Most minority ethnic people are employed in farming (75.1%), and a wage from a formal contract outside of farming is fairly uncommon (8.1%) [58].

As well as some of the poorest communities, farming communities are often located in precarious environments prone to extreme climate events or gradual sea level rises [59]. Employment in tourism is increasing, but is highly seasonal, temporary, low-paid, and common in low-income households [60]. Vietnam, like other fast-growing economies in Asia, has experienced high graduate unemployment as low as 27% in 2014 [58]. Earlier studies have indicated that 60% of graduates in Vietnam are unemployed or have insecure employment, with poor working conditions, job instability, low wages, and job informality being common issues for young people [61, 62].

### Two wave study

**Fig 2:**
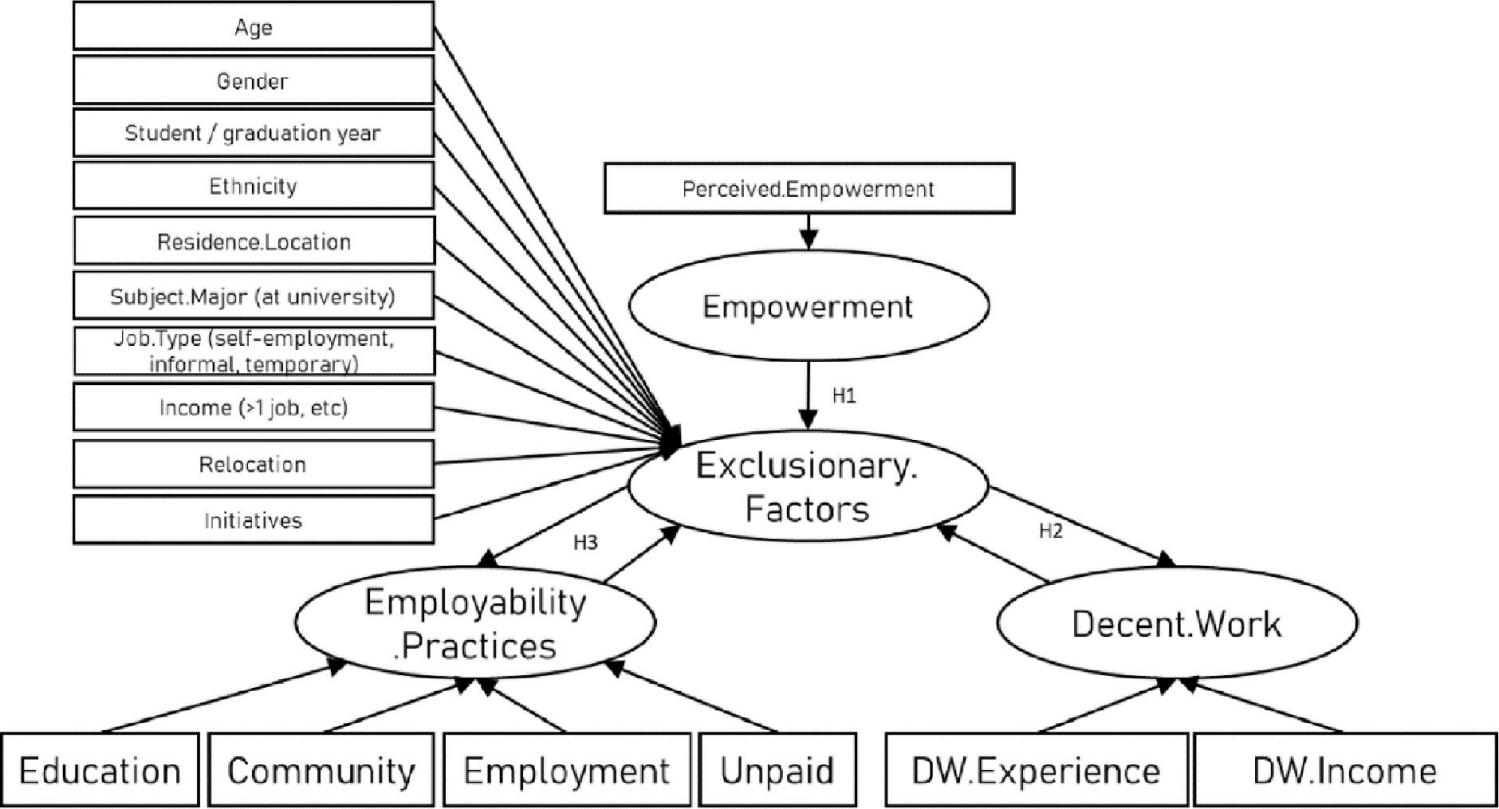
Hypothesized relationships in this study.

This study involved two waves to examine the conceptualisation of the relationship between empowerment, expression of this in contexts, and experience of Decent Work (Fig 1). The first tested the operationalisation of the model in the hypothesised model perspective of minority ethnic minority young people aged 18–25 (proposed in Fig 2), and the second from the perspective of stakeholders experienced in the hiring or placement of minority ethnic young people in work (i.e. employers, university careers and placement staff, and policymakers). As such, the two waves provide a perspective triangulation strategy to strengthen the validity of the findings in relation to the target population.

### Instruments

A survey instrument was derived from the literature outlined above, using a Likert scale ranging from 1 (totally disagree) to 5 (totally agree). An English version was triple reverse translated between English and Vietnamese by three bilingual researchers before it was then piloted with 30 individuals across the target population. The instruments needed to be articulated in ways which were understandable to participants where literacy could not be guaranteed across all three study sites. Following the pilot, the surveys were revised for internal/construct validity. In addition to basing the survey items on previously validated constructs through the robust translation and piloting processes above, several reliability tests were employed; in the pilot, a Cronbach alpha of 0.900 was achieved, above the 0.7 threshold [63]. For the minority ethnic young person’s survey, 50 of the 70 items had a factor loading of >0.6 and so were retained for further analyses. The Cronbach’s Alpha Coefficient was 0.900 for all 50 variables, which indicates that the instrument had a good internal consistency. Moreover, Cronbach’s alpha values for each construct were above this acceptable level (0.70): Employability Empowerment 0.934, Decent Work 0.823, and Contextualised Employability Practices 0.712. For the stakeholder survey, the Cronbach’s alpha was 0.977 indicating good reliability [63]. The Cronbach’s alpha values for each construct were also above the acceptable level (0.70): Employability Empowerment 0.803, Decent Work 0.795, and Contextualised Employability Practices 0.887.

The surveys were administered in three different sites of Vietnam, including North and South mountainous provinces as well as central urban conurbations. These sites covered a diversity of communities including farming and non-contracted employment conditions (in mountainous areas), as well as those less dependent on farming and more oriented towards waged and self-employment opportunities (urban areas). The surveys were administered face to face with the research team with support from minority ethnic research assistants who could speak local languages (other than Vietnamese). While Vietnamese was the dominant language at the three sites, having multi-lingual research assistants at each site meant that the research team could explain any nuances of the survey questions across multiple languages at each site if and when required. Evidence also indicates that this approach builds trust and therefore reliability of responses [64].” Participants were recruited and surveys administered between the 1^st^ to the 30^th^ June 2020. Informed consent was given in written and oral format. Ethical review and institutional permission were granted at a UK institution with input from Vietnamese researchers.

### Measurement

First, data were analysed with descriptive statistics to summarise patterns [65], and second, Structural Equation Modelling (SEM) was deployed to depict relationships among observed variables to provide a quantitative test of a theoretical model hypothesised from the extant literature [66]. SEM enabled a complex model to be examined [67], using SPSS AMOS Version 27 to specify, test, and modify models. Before testing the statistical model, we conducted a succession of Exploratory Factor Analysis (EFA) and Confirmatory Factor Analysis (CFA) procedures. EFA was primarily used for data reduction through the exploration of responses and CFA was primarily used for the confirmation and measurement theory [68]. All factor loadings within the data set applied to the SEM were more than the recommended threshold of 0.50 [69].

A CFA was conducted to examine whether the primary concepts examined in this study (see Figs 1, 2 and 4) were distinct constructs [68]. To do this, we first examined a measurement model that included all measures. We then compared the measurement model to similar models that set the factors to correlate at 1.0 to keep the basic measurement model structure comparable, allowing for meaningful Chi-square difference tests. Furthermore, to test the measurement model, the CFA was conducted using the Maximum Likelihood (ML) method, which is the most widely used method for parameters estimation in SEM [70]. To improve the measurement model goodness-of-fit, several modifications were introduced to the first-run model. In order to improve the model fit, we correlated parameter errors that are part of the same factor [71]. Therefore, different parameter errors were correlated to improve the overall model fit. In addition, through experimentation, specific items were removed from the models (these are reported below).

SPSS AMOS version 27 generates 25 different goodness-of-fit measures and the choice of which to report is a matter of dispute among methodologists [72]. However, it is recommended to report Chi-squared statistics in addition to another absolute index such as RMSEA and an incremental index such as CFI, and when comparing models of varying complexity, it is recommended to add Parsimony-Adjusted Measures (PNFI) [72]. Despite there being no consensus towards which GFI reports to present, the following indices should however, be reported when presenting model fit: 1) The model chi-square 2) RMSEA 3) CFI and 4) SRMR [72].

## Study 1: Survey of minority ethnic young people

### Variables

In study 1, the conceptualisation of the study (Fig 1) was operationalised with a set of hypotheses derived from the literature (Fig 2).

*Empowerment employability*. This construct was operationalised with different expressions of power (over, with, within, to) across a social system. Through translation and piloting processes, this final construct was represented through 15 items in Vietnamese (supported with immediate explanatory guidance to assure construct validity). Examples of the items in English translated to: *“I believe I can change and improve the way I earn a living or my employability”*, *“I use my networks to help improve how I earn a living or my employability”*, *and “I make links between different settings to help me improve how I earn a living or my employability”*.

*Exclusionary factors*. Exclusionary factors included: demographics in terms of age, gender, and ethnicity; location and relocation; job type in terms of work with contract, without contract (informal), and self-employment; subject major; student/graduation year; income sources (none, one, more than one); and policy initiatives (national and local vocational education, financially incentivised access to university, removal of examination requirements, and specialist boarding schools and foundation programs).

*Contextualised employability practices*. Three main contexts where students and graduates develop their employability were derived from the extant literature, namely education leading up to and including education, family as an embedded community setting, and through employment. Items were derived from the employability empowerment items but contextualised with examples for that context. Through translation and piloting processes, the final construct was represented through 15 additional items in Vietnamese across the three contexts.

*Decent Work*. The main dimensions of decent work were derived from the original and psychological definitions. Through translation and piloting processes, this final construct was represented through 15 items in Vietnamese (supported with immediate explanatory guidance to assure construct validity). We adopted a more culturally derived understanding of Decent Work, which acknowledges that some concepts may not be locally meaningful. For example, the Decent Work item related to health care provision was removed as this is not found to be typical or meaningful in Vietnam and so did not make contextual sense to participants. Examples of the items in English translated to *“I work in a place(s) where… I can depend on my work for a regular income”*, *“…where I am free to express my concerns at work”*, *“…where there is equality of opportunity and treatment for all ethnicities”*, *and “…where the work is satisfying”*.

### Sample

Study 1 focused on minority ethnic young people aged 18–25 across (N = 1071), summarised in Tables 1 and 2. The sample was spread more or less evenly across ages 18–25 and genders, and was comprised of 42 ethnic groups including populations smaller than 1000. Most respondents were students, and a wide range of subject majors were represented in the study. Most respondents reported no income from work and were currently unemployed (but had experienced a form of work), or were in a form of permanent, temporary or informal employment. In terms of geographies, most respondents were in the North Mountain centre or the South centre, but there was representation from rural and urban areas surrounding the three research sites (north, central and south). A probability sampling method was used given the difficulty and sensitivity of reaching minority ethic groups in Vietnam, an approach which allowed for potentially everyone from the population an opportunity to be selected. Here, the sampling process involved (1) selecting three sites which represented the three population zones of urban, rural and mountainous areas where most minority ethnic groups live and study (validated by Project Advisory Group which involved government officials overseeing minority ethnic groups in Vietnam), (2) getting permission and validation from the peoples’ committee of minority ethnic groups across the three sites, (3) selecting institutions from the three sites which are recognised by government and peoples’ committees to have some of the highest proportion of ethnic minorities) (4) inviting selective employers who have businesses located at 3 sites and employ minority youth for surveys and interviews, (5) sending the research information to the institution leaders to get their permissions, (6) once the institutions approved, the research information was sent to the students and graduates by the institutions for participant recruitment on the voluntary basis, and then (7) the research team and multi-lingual research assistants visited the sites and conducted the interviews ensuring that the participants met the inclusion criteria (that they are current or recent student (as above), had experienced a form of formal or informal work, and identify other than the Kinh group).

**Table 1:** Description of sample (I): Age, gender, location, income and type of work.

Age	Frequency	%	Location	Frequency	%	Income	Frequency	%
18	106	10%	North mountain center	559	52.2%	No income from work	725	67.7%
19	144	13%	South center	270	25.2%	Income from one source of work	313	29.2%
20	164	15%	South mountain area	132	12.3%	Income from 2–3 sources of work	29	2.7%
21	176	16%	North center	108	10.1%	Income from more than 4 sources of work	4	0.4%
22	161	15%	Abroad	2	0.2%	**Total**	**1071**	**100.0**
23	104	10%	**Total**	**1071**	**100.0**			
24	77	7%				**Type of work**	Frequency	%
25	139	13%	**Home and work location over the last 7 years**	Frequency	%	Currently unemployed (but had experienced work)	488	45.6%
**Total**	**1071**	**100.0**	Different	833	77.8%	Employed with permanent contract	294	27.5%
			Same	238	22.2%	Employed with temporary contract	124	11.6%
**Gender**	Frequency	%	**Total**	**1071**	**100.0**	Informal work (without contract)	98	9.2%
Women	613	57%				Self-employed	67	6.3%
Men	458	43%				**Total**	**1071**	**100.0**
**Total**	**1071**	**100.0**						

**Table 2:** Description of sample (II): Ethnicity, student/graduate status, major, and education type.

Ethnic group	Frequency	%	Student status	Frequency	%
Thai	204	19.0%	1^st^ year student	109	10.2%
Hmong	152	14.2%	2^nd^ year student	199	18.6%
Tay	111	10.4%	3^rd^ year student	206	19.2%
Co Tu	85	7.9%	4^th^ year student	163	15.2%
Kinh (Mixed/Other)	64	6.0%	5^th^ year student	10	0.9%
Nung	61	5.7%	6^th^ year student	22	2.1%
Dao	48	4.5%	Graduate	362	33.8%
Muong	48	4.5%	**Total**	**1071**	**100.0**
Pa co	44	4.1%	**Major**	Frequency	%
E de	43	4.0%	Others	562	52.5%
Giay	32	3.0%	Education	193	18.0%
Gia rai	23	2.1%	Economics	160	14.9%
Ta oi	18	1.7%	Agriculture	91	8.5%
Ha Nhi	13	1.2%	Engineering	47	4.4%
Ka dong	13	1.2%	Business	18	1.7%
Cham	13	1.2%	**Total**	**1071**	**100.0**
Giay	10	0.9%	**Training type**	Frequency	%
Bru-Van Kieu	9	0.8%	University	681	63.6%
Kor	9	0.8%	Vocational college	290	27.1%
San Diu	8	0.7%	Lower Vocational College	100	9.3%
Mạ	8	0.7%	**Total**	**1071**	**100.0**
Others*	7	0.7%			
Ko ho	7	0.7%			
Ta Rieng	6	0.6%			
Han	6	0.6%			
Cao Lan	4	0.4%			
Xe dang	3	0.3%			
Chu ru	3	0.3%			
Tho	3	0.3%			
Bana	2	0.2%			
Kho mu	2	0.2%			
Xa Pho	2	0.2%			
Xa *p*ho	2	0.2%			
Mạ	2	0.2%			
La Chi	1	0.1%			
Hre	1	0.1%			
Pa di	1	0.1%			
Sila	1	0.1%			
Xo dra	1	0.1%			
Lao	1	0.1%			
**Total**	**1071**	**100.0**			

* Pu peo, Lu, Lo lo, La Hu, Bo–Y–these groups have a very small number of population (under 1000)

### Study 1 Results

**Fig 3:**
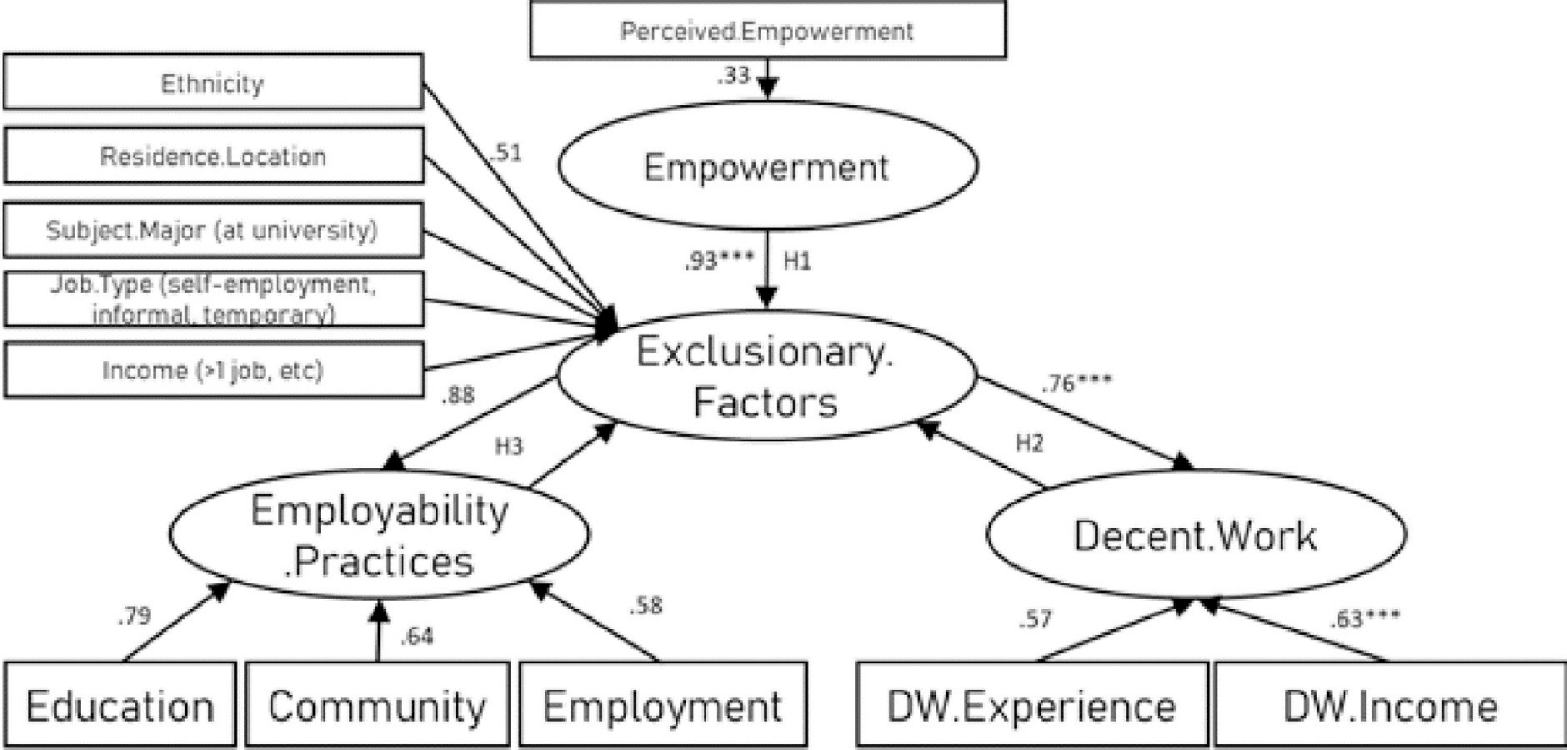
Minority ethnic young person perspective (model with GFI, CFI, 0.951).

The EFA identified 8 factors which explained 71.59% of the variance. The model was refined through an iterative process to identify the strongest goodness-of-fit model (Fig 3). In line with the GFI reporting conducted by Kline [72] the model fit data showed that our hypothesised measurement model fits the data very well, χ2 (201, N = 1071) = 468.11, p < 0.000, CFI = 0.951, IFI = 0.943, RMSEA = 0.05 and SRMR = 0.07. A variety of items were removed from the model to enhance goodness-of-fit, in terms of (1) exclusionary factors: age, student/graduate status, gender, relocation, and policy initiatives, and (2) in terms of contextualised employability practices, the item “*To find unpaid work experience*” in Education and Community, and Employment settings were removed and the item “*To learn new technology skills*” were removed from Community and Employment settings. Analyses are presented in Tables 3 and 4.

**Table 3:** Presents the means, standard deviations, correlations and coefficient alphas (α) among the study variables. As shown in Table 4, all hypothesized pairs of relationships were statistically significant.

Means, Standard deviation, reliabilities and intercorrelations among study variables							
Variables	M	SD	1	2	3	4	5
1) Employability Factors	3.07	0.79	-0.83				
2) Decent Work	3.39	0.65	0.84**	0.88			
3) Exclusionary Factors	4.38	0.87	0.50**	0.65**	-0.73		
4) Empowerment	4.36	0.82	0.71**	0.72**	0.66**	-0.81	
5) Demographic	4.36	0.79	0.50**	0.36**	0.36**	0.54**	-0.81

*N* = 1071. Coefficient alphas are listed in parentheses along the diagonal

*p < 0.05

**p < 0.01 (2-tailed)

**Table 4:** Shows the overall results of the multiple regression analysis for collapsed variables. VIF and Tolerance values shown in the output tables suggest that there is no multicollinearity issue. The final model achieved where all the predictor variables were significant I.e. p < .05.

	Unstandardized Coefficients		Standardized Coefficients			Collinearity Statistics	
Model	B	Std. Error	Beta	t	Sig	Tolerance	VIF
	(Constant)	-822	2.56	-3.215	0.001		
	Employability Factors	319	0.67	4.773	0.000	0.59	1.695
	Decent Work	297	0.46	6.482	0.000	0.962	1.039
	Exclusionary Factors	160	0.74	2.174	0.03	0.645	1.551
	Empowerment	250	0.79	3.153	0.002	0.687	1.456

## Study 2: Survey of stakeholders

### Variables

**Fig 4:**
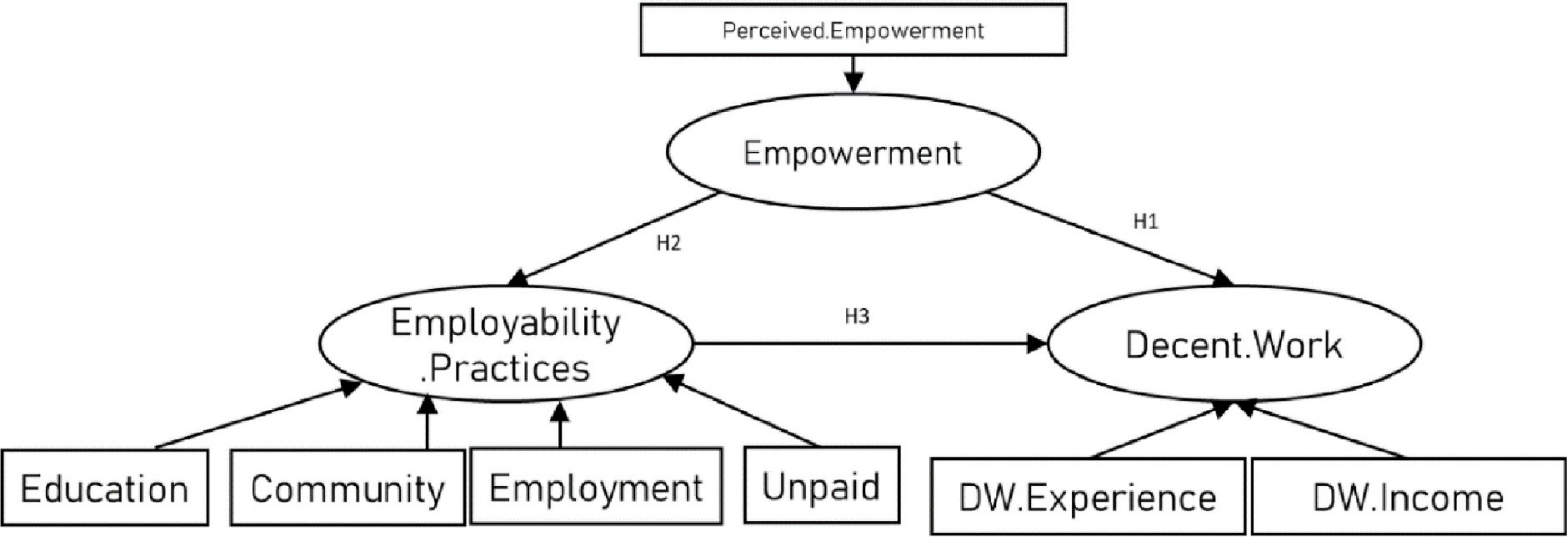
Hypothesized relationships from stakeholders’ perspectives.

Study 2 focused on the stakeholder perceptions of minority ethnic young people in terms of empowerment and the expression of their employability as defined in Survey 1. This includes policymakers, employers, and careers professionals involved in the recruitment and employment of minority ethnic young people. Employability empowerment, contextualised employability practices across three main contexts, and Decent Work were defined and operationalised as described in Study 1 but were worded from the perspective of stakeholders with reference to minority ethnic young people aged 18–25 (Fig 4).

### Sample

Study 2 examined the perception of stakeholders who work closely with minority ethnic young people in the three locations of the overall study (N = 204). The sample is summarised in Table 5. Most respondents were the dominant ethnic group, and most were in northern provinces (e.g. Hanoi 24.5%) or central provinces (e.g. Hue 16.7%). This distribution reflects the distribution of institutional policy makers and employers and their representatives in the north and centre of Vietnam.

**Table 5:** Description of stakeholder sample: Ethnicity and location.

Ethnic group	Frequency	Percent	Location	Frequency	Percent
Kinh	136	66.7%	Hanoi	50	24.5%
Tay	10	4.9%	Hue	34	16.7%
Co Tu	9	4.4%	Son La	26	12.7%
Undisclosed	6	2.9%	Quang Nam	26	12.7%
Muong	6	2.9%	Lai Chau	16	7.8%
Mong	5	2.5%	Lao Cai	16	7.8%
Thai	5	2.5%	Thai Nguyen	9	4.4%
E De	5	2.5%	Bac Kan	9	4.4%
Nung	4	2.0%	Khong	5	2.5%
Pa Co	4	2.0%	Daklak	5	2.5%
Ta Oi	3	1.5%	Vinh Phuc	2	1.0%
Mnong	2	1.0%	Phu Thọ	1	0.5%
Bru Van Kieu	2	1.0%	Dien Bien	1	0.5%
San Diu	1	0.5%	Quang Trị	1	0.5%
Ha Nhi	1	0.5%	Hoa Bình	1	0.5%
Hoa	1	0.5%	Thanh Hoa	1	0.5%
Gia Trieng	1	0.5%	Tay Nguyen	1	0.5%
Xieng	1	0.5%	Total	204	100
Dao	1	0.5%			
Pahy	1	0.5%			
Total	204	100			

### Study 2 Results

**Fig 5:**
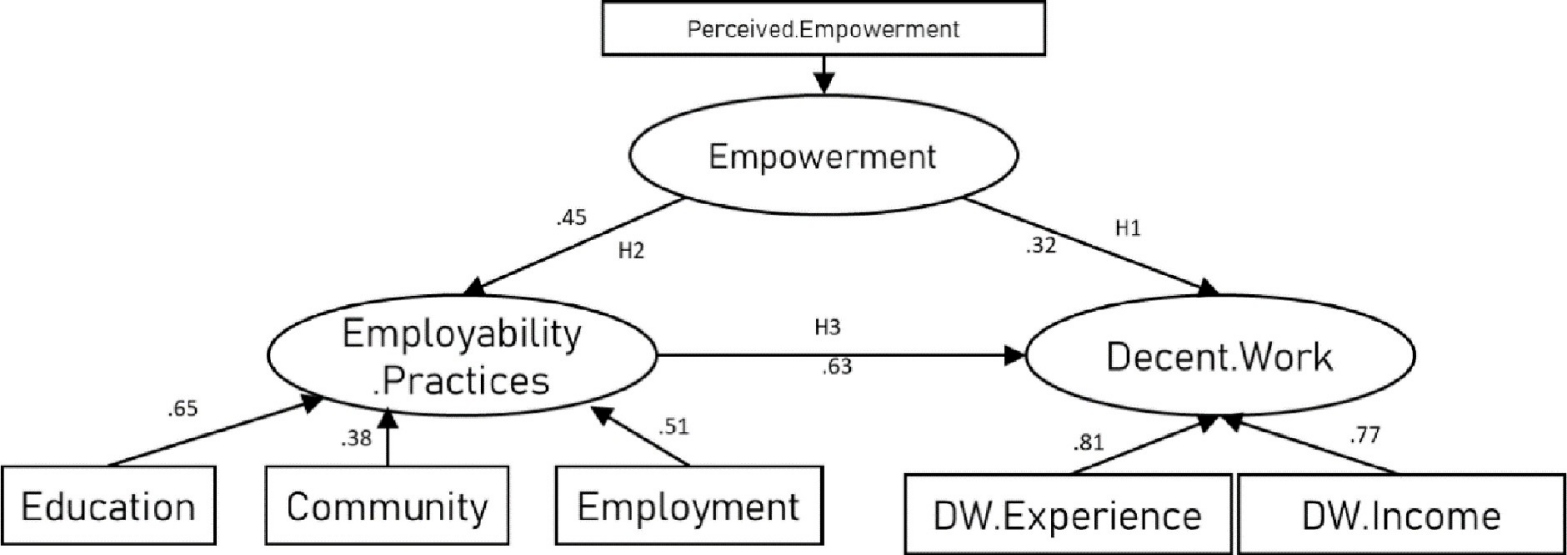
Stakeholder perspective on role of empowerment (model with GFI, CFI—.0.918).

The EFA identified 15 factors which explained 73.12% of the variance. The model was refined through an iterative process to identify the strongest goodness-of-fit model (Fig 5). In line with the GFI reporting conducted by Kline [72] the model fit data showed that our hypothesised measurement model fits the data well, χ2 (348, N = 204) = 1082.00, p < 0.000, CFI = 0.918, IFI = 0. 943, RMSEA = 0.056 and SRMR = 0.061. Three items related to minority ethnic young people seeking or taking unpaid work opportunities in the three settings were removed from the model to enhance model goodness-of-fit. Analyses are presented in Tables 6 and 7.

**Table 6:** Presents the means, standard deviations, correlations and coefficient alphas (α) among the study variables from the stakeholders perspective.

Variables	M	SD	1	2	3	4
1 Empowerment	3.42	0.72	0.72**	0.73**		
2 Decent Work	3.41	0.8	0.50**	0.37**	0.36**	
3. Employability Factors	4.38	1	0.64**	0.65**	-0.32	-0.81

*N* = 204. Coefficient alphas are listed in parentheses along the diagonal *p < 0.05, **p < 0.01 (2-tailed)

**Table 7:** Coefficient analysis presents the overall results of the multiple regression analysis for collapsed variables. VIF and Tolerance values shown in the output tables suggest that there is no multicollinearity issue.

	Unstandardized Coefficients		Standardized Coefficients			Collinearity Statistics	
Model	B	Std. Error	Beta	t	Sig	Tolerance	VIF
(Constant)	-1232	0.213		-5.795	0.000		
Empowerment	184	0.67	165	2.745	0.006	0.328	3.045
Decent	326	0.48	245	6.803	0.000	0.91	1.099
Employability Factors	469	0.59	328	7.878	0.003	0.684	1.461

## Discussion

This study directly responds to calls to redress imbalances in the study Decent Work, providing alternatives to studies focused on the psychology of individuals in developed countries who work in typical work arrangements. Drawing from sociological perspectives, this research hypothesised the mediating role between spatialised exclusionary factors and (H1) the relationship between perceived employability empowerment and experience of Decent Work, (H2) the relationship between perceived employability empowerment and the expression of practices in different settings, and (H3) the relationship between contextualised employability practices and experience of Decent Work. Our analysis finds support for these relationships, from the perspective of minority ethnic young people and the stakeholders involved in their recruitment and employment. These findings, in part, echo the extant literature in terms of the possibility of psychosocial resources to navigate the wider economic labour market forces which predict Decent Work [11]. This study extends those findings through the notion of employability empowerment which integrate broader, sociocultural resources that are potentially negotiational in the sense they embody abilities to change circumstances [6, 7, 48]. This broader conceptualisation offers an alternative perspective to how people, including those who typically possess relatively limited resources such as young ethnic minority students and graduates, can mediate, that is, change or improve, their livelihood circumstances. This finding also sits in contrast to the predominant literature Decent Work to argue that navigational and negotiational resources do not just mediate experiences of Decent Work, but are, themselves, entangled in localised sociocultural schemas.

This study extends existing evidence to suggest that ethnicity remains a predictive marginalising factor in accessing Decent Work even when factoring in a broader concept of employability empowerment. This finding expands the role of critical consciousness in education beyond skills acquisition and generation [73], but also provides empirical evidence as to its boundary constraints in sociocultural settings. Moreover, the predictive value of ethnicity is strongest when intersecting with location and other variables which would be interpreted spatially in local labour markets, namely, educational subject major, form of employment and sources of income. According to the findings of this study, then, the effects of ethnicity are composite and at least in part, spatial, reflecting local sociocultural contexts, and shaping the ways in which employability empowerment is expressed across settings to influence experiences of Decent Work.

Such a perspective amplifies the possibility of more deeply contextualised research into Decent Work and the potential of more nuanced perspectives across sociocultural landscapes. While there may be an emerging departure from the dominant normative approach to examining Decent Work through the unification of individual psychological instruments [15, 16], a spatialised approach suggests that marginalising factors may not just manifest differently across countries but may manifest differently across geographic or spatial areas which have different sociocultural characteristics such as industries or access to infrastructural services. This aligns with the notion of sociocultural schemas as a function of place [27], which helps to explain spatial differences in marginalisation across a single country.

However, in contrast to the extant literature, there were spatialised exclusionary factors and items related to the expression of empowerment through contextualised employability practices which reduced the predictive value of the models examined. In terms of spatialised exclusionary factors, four items reduced the relative predictive value of the models examined: gender, age, student / graduate status, relocation, and policy initiatives. Gender is perhaps the most surprising of these items, given the relatively long-standing and strong evidence base of gender discrimination in workplaces [74], its predictive role as a marginalising factor in Decent Work [11], and its spatialised variation in the effects it creates across locations and organisations [27]. There is some emerging evidence which identifies scenarios where there are no statistically significant differences between women and men in terms of job satisfaction and work-life balance, for example, the case of highly educated, trained, and experienced physicians in China [75].

Sociologically, one explanation of why gender may not be as predictive as expected could relate to the way intersectional identities aggregate to create differential effects on discrimination and associated employment outcomes. Exclusionary factors such as gender and ethnicity (as well as age) can lead to additional effects (“additive”), multiplicative effects where the addition of categories amplify negative effects (“amplified congruence”) or have no additional effects because of perceived similarity (“muted congruence”) [76].

The findings of this study suggest that the addition of gender to ethnicity as a predictor of Decent Work–when factoring in the role of employability empowerment for this population and in an emerging economy with graduate oversupply–may therefore display a muted congruence effect. In other words, with a sense of empowerment (or lack thereof), ethnicity is sufficient to predict experience of Decent Work because of a perceived congruence to gender in discriminative processes [77]. This reflects other research into deprivation in Vietnam where location was a greater predictor of all forms of deprivation [57] and where gender discrimination is less prominent in some rural areas relative to urban areas [78]. This discussion could also be recited in relation to explaining why age decreased the predictive utility of the model [33]. However, the study sample is limited to students and graduates aged 18–25 which might explain its relatively limited impact in the model examined.

It is possible that the respective role of ethnicity might be pronounced in the research context of this study given the high diversity of minority ethnic groups (there are 54 recognised ethnic groups in Vietnam) [59]. Though it is important to recognise that this study measured the effects of a spatialised notion of ethnicity, that is ethnicity is measured alongside location. Research has already indicated that the visibility and institutional or group-level recognition of ethnic groups in sociocultural and geographic locations can vary considerably [29]. More recently, however, evidence into discrimination in multi-ethnic communities has indicated that inter-group contact and institutional arrangements for minority groups at the local level can influence the extent and nature of racial discrimination [79]. As our study included mountainous areas which are historically and typically characterised by ethnic minority groups [59] as well as urban areas where discrimination has been evidenced to be more common [78], we believe our results provide a representative picture of spatialised factors. This means we believe that the geographic diversity of our sample provides strong empirical justification for a spatialised notion of ethnicity and how it can shape Decent Work.

At the same time, as our research utilised subjective measures related to Decent Work (e.g. self-reports of income fairness rather than absolute income levels), it is also possible that the model is masking differential expectations between gender identifications. There is now a long-standing evidence base that societal and occupational norms shape the self-efficacy beliefs, familial and work role expectations, and employment expectations of women including from Vietnam [78]. Here, such subjective beliefs and expectations frame and shape responses from a marginalised or disadvantaged position (relative to a majority group), thereby making it more difficult to ascertain absolute comparisons. Recent empirical work has highlighted this phenomenon with workers engaged in precarious work environments, where they were observed expressing an appreciative disposition towards their precarious circumstances rather than a critical evaluation of conditions that would otherwise be described as abhorrent [80].

This research posited that employability empowerment is expressed through contextualised employability practices in education [39], community [81], and employment [52], which then shapes engagement with Decent Work. Whilst this reflects prior empirical work, there were two main items that were excluded from the model. The first relates to the utilisation of those contexts to find unpaid work, which when included in the model weakened the predictive value of the models in both studies (i.e. from both the minority ethnic student and graduate and the stakeholders involved in their recruitment and employment). This might reflect the awareness, perceived relative attractiveness, and uptake of unpaid work opportunities available through those settings. Evidence suggests that ‘not-for-credit’ opportunities associated with enhancing employability such as business start-up or volunteering can be perceived as some of the least valuable in terms of developing new skills, experience, networks, and employment [82]. However, paradoxically, the relative role of engaging in unpaid work might also be an indicator of a greater sense of employability empowerment. Here, research has found that when students and graduates have a greater sense of self-efficacy, control, and perceived employability, it can reduce their proactive behaviours such as career planning and development [83].

The second item excluded from the model related to the utilisation of community and employment contexts to “learn new technology skills” (the same item in the education context did not meet factor loading criteria for inclusion through model measurement procedures). This might reflect two separate phenomena. The first is that technology is so pervasive to the sample group (i.e. ethnic minority students and graduates aged 18–25) that it is no longer perceptible as ‘new technology’ and rather a habituated practice that is not reified as a categorically different code. The second, conversely, is that the sample may perceive they do not have access to new technology across settings or are not aware of the possible importance to employment outcomes. Given the relatively low levels of engagement with technology and technology skills development across education, community, and employment, especially in rural areas [56], we are more convinced by the second explanation. According to the model measured in this study, there are relatively more useful factors that predict Decent Work for the sample population, including employability empowerment, the expression of this across settings, and the mediating effects of location, ethnicity, subject major, job type, and sources of income.

## Limitations, boundaries, and future research

The conceptual boundaries of this study were derived from empirical work conducted in developed countries in typical employment contexts, but this research is contextualised within one of the fastest growing, emerging economies in the world and with some of the most detrimentally impacted by precarious work. It was conducted during a global pandemic which might reasonably shape the responses offered by participants (although Vietnam was not in lockdown during the data collection period). For example, the threat of lockdown might have accentuated the familial or community context over employment contexts or undermined and therefore underestimated the sense of employability empowerment due to a diminished sense of control compared to pre-pandemic times [20]. Despite the context in which the data was collected, the hypothesised model retained a statistically good fit. It is possible that this study is temporally located, for example, in terms of levels of graduate oversupply [84], and further time-series studies across time or across life spans could build the generalisability of the findings.

Unlike the psychological study of Decent Work, this study has introduced a more sociologically oriented notion of employability empowerment which is mediated by the spatialised exclusionary factors of specific locations. Employability empowerment, as navigational and negotiational in character, is not an individual characteristic which is more or less stable, and so is likely to be variable across spatialised settings and time. The levels of discrimination will differ across these settings, partly determined by the level and historical context of inter-group contact and the institutional recognition and status given to minority ethnic communities at the local level [79]. Similarly, it is important to recognise the population for this study has been ethnic minority students and graduates aged 18–25, and results should be bounded respectively. With this sample, age or student/graduate status were not significant mediating factors in our model, but future research should examine the spatialisation of Decent Work with a wider age range. It is reasonable to assume that across spatialised settings and life spans, ethnicity, gender, and age, may variously interact and create differential effects, from muted congruence, additive, or multiplicative effects [76]. Therefore, undertaking further large-scale studies across other countries with different sociocultural geographies and demographics might generate further insights into how employability empowerment interacts with the exclusionary factors in relation to Decent Work, especially as societies age.

## Implications

A spatialised approach to Decent Work which recognises the role of employment empowerment implicates institutions operating across education, community, and employment settings. It is one which is sensitive to the possibility that initiatives may well accelerate employment integration (e.g. the achievement of a job), but that such initiatives can also promote the acceptance of job terms and conditions (such as wage) lesser than they would have been through other means [37]. For education, our research challenges generalised or ‘whole institution’ approach to employability skills matrices and demands a more sophisticated sensitivity to localised labour markets [39]. It is likely that this will reflect the positioning of educational institutions, such as locally, regionally, nationally, or internationally oriented universities and their respective target labour markets. Such sensitivities are likely to include a range of interventions to reflect the specific needs of locations, subject areas, and ethnic groups [3]. According to the model presented here and recent research which indicates how students and graduates perceive ‘for-credit’ opportunities [85], the integration of vocational and work-based ‘for-credit’ opportunities as part of courses, and which are connected to workplaces, are particularly promising if they focus on dimensions of employability empowerment.

Governmental policy frameworks and initiatives would benefit from the same locational principles in encouraging Decent Work. The policy initiatives examined through this study have created employment outcomes for ethnic minority groups [56], but they did not provide predictive utility in relation to Decent Work. This may be related to Vietnam’s emerging, quasi-decentralised governance framework and the resulting variability of implementation at the local level [86]. National level coordination of localised priorities and action plans could provide a strong platform for the realisation of Decent Work across provinces, as young people do move to find work. Policy might incentivise the development of employment empowerment for minority ethnic students and graduates, such as the subsidisation of targeted upskilling opportunities through community organisations and employers which focus on developing the dimensions of employment empowerment in particular vocational fields. This echoes other policy approaches internationally, for example the tax levy introduced to encourage UK employers to upskill their workforces through apprenticeships in priority areas but builds on this with specific nuance. Given the dynamic nature of labour markets over time, such implications would provide a stronger platform to better enable minority ethnic young people to engage in Decent Work across geographic locations.
